# FXYD6 overexpression in HBV-related hepatocellular carcinoma with cirrhosis

**DOI:** 10.1515/biol-2020-0027

**Published:** 2020-04-20

**Authors:** Xiongfei Chen, Lishuang Ding, Deshuai Kong, Xiulei Zhao, Lili Liao, Yaomin Zhang, Fengshan Li, Ruhai Liu

**Affiliations:** Department of Hepatobiliary and Pancreatic Surgery, Cangzhou Central Hospital, 16 Xinhuaxi Road, Yunhe District, Cangzhou, 061001, P. R. China

**Keywords:** FXYD6, hepatocellular carcinoma, hepatitis B virus, microvascular invasion, early recurrence

## Abstract

**Objective:**

The aim of this study was to investigate the expression of FXYD domain-containing ion transport regulator 6 (FXYD6) mRNA and protein in hepatitis B virus (HBV)-related hepatocellular carcinoma (HCC) tissues with cirrhosis, the corresponding paracancerous tissues and the normal liver tissues, and to explore the clinical significance of FXYD6 expression in HBV-related HCC with cirrhosis.

**Methods:**

The FXYD6 mRNA and protein were examined by semi-quantitative reverse transcription polymerase chain reaction and immunohistochemistry, respectively.

**Results:**

The FXYD6 mRNA in HBV-related HCC tissues was significantly higher than that in the cirrhosis tissues or that in the normal liver tissues. The positive expression rate of FXYD6 protein was statistically higher in HBV-related HCC tissues than that in HBV-related cirrhosis or that in normal liver tissues. There was no significant correlation between the expression of FXYD6 protein and gender, age, histological differentiation, tumor diameter, tumor number, integrity of tumor capsule or not and alpha fetoprotein (AFP) concentration in serum, but the protein expression was associated with microvascular invasion, pathological stage, and early recurrence after operation within 1 year.

**Conclusion:**

FXYD6 might be involved in hepatocyte carcinogenesis and tumor progression in HBV-related HCC with cirrhosis and indicated a poor prognosis.

## 1 Introduction

Hepatocellular carcinoma (HCC), the most common of the hepatobiliary malignancies, is ranking fourth among the causes of malignant tumor death in the world and closely related to cirrhosis with hepatitis B virus (HBV) infection in Asia [1,2]. Radical resection is still the most effective treatment for HCC at present, but usually intrahepatic metastasis through microvascular invasion (MVI) in early stage occurs, which leads to frequent recurrence post operation [3]. Thus, it is imperative to further explore the mechanism underlying the occurrence and metastasis of HCC and to find a therapeutic target associated with MVI in this malignant disease.

FXYD domain-containing ion transport regulator 6 (FXYD6), an ion channel-associated transmembrane protein, is an important regulator of Na, K-ATPase [4]. It is highly expressed in brain tissues and plays an important role in the development and excitability of neurons [5,6]. In addition, it is a tumor-associated protein, highly expressed in various tumor types, as reported in our previous studies [7,8], and involved in proliferation and metastasis of HCC cells and osteosarcoma cells [8,9,10]. However, the expression of FXYD6 mRNA and protein in HBV-related HCC with cirrhosis and the relationship between the protein expression and clinicopathological features in HCC remain elusive. The aim of this study was to investigate the expression of FXYD6 mRNA and protein in the malignant disease, paracancerous cirrhosis, and normal liver tissues and to analyze the relationship between FXYD6 expression and clinicopathological features including MVI and early recurrence.

## 2 Materials and methods

### 2.1 Clinical samples

Thirty-five fresh HBV-related HCC tissues with cirrhosis, 30 fresh cirrhosis tissues adjacent to HCC, and 10 normal fresh liver tissues distal to the surgically resected hepatic hemangioma without HBV infection were collected from the Department of Hepatobiliary and Pancreatic Surgery in Cangzhou Central Hospital (Cangzhou, China) between March 2014 and March 2018. The fresh tissues were stored in liquid nitrogen within 30 minutes after harvest and subsequently stored in a refrigerator at −80°C.

In addition, the formalin-fixed and paraffin-embedded tissue samples were obtained from 52 HBV-related HCC patients with cirrhosis who underwent radical surgery in the hospital between March 2012 and March 2017. HCC was staged according to the eighth edition of the HCC AJCC staging system. None of the patients received preoperative chemotherapy, radiotherapy, or biotherapy. Twenty-eight cirrhosis tissues matched with the primary tumor, and 15 normal liver tissues distal to the hepatic hemangioma in our hospital were also studied. Patients’ information, including gender, age, differentiation, tumor diameter, tumor number, integrity of tumor capsule or not, MVI, pathological stage, and AFP concentration in serum, was recorded at the hospital.

The mean age of the patients with HCC (44 males and 8 females) was 55.2 years, ranging from 30 to 78 years, and the median age was 56.5 years. Seven neoplastic specimens were well-differentiated carcinomas, 24 were moderately differentiated carcinomas, and 21 were poorly differentiated carcinomas. There were 22 patients with tumor 5 cm or less in diameter and 30 patients with tumor larger than 5 cm in diameter, 40 patients with single tumor and 12 patients with multiple tumors, 31 patients with integral tumor capsule and 21 patients with incomplete tumor capsule, 21 patients with MVI and 31 patients without MVI, 33 cases in stage I–II and 19 cases in stage III, and 38 cases with AFP < 400 ng/mL and 14 cases with AFP ≥ 400 ng/mL. The patients were reviewed at intervals of 1–3 months after surgery and recorded whether the carcinoma relapsed within 1 year. The patients received no treatment before recurrence.


**Informed consent:** Informed consent has been obtained from all individuals included in this study.
**Ethical approval:** The research related to human use has been complied with all the relevant national regulations, institutional policies, and in accordance with the tenets of the Declaration of Helsinki and has been approved by the authors’ institutional review board or equivalent committee.

### 2.2 Semi-quantitative reverse transcription polymerase chain reaction (RT-PCR) for FXYD6

Total RNA was isolated from tissue samples using TRIzol reagent (Thermo Fisher Scientific, Inc., Waltham, MA, USA) according to the manufacturer’s protocol. cDNA was synthesized from 2 µg total RNA using an EasyScript Plus™ cDNA Synthesis Kit (ABM Inc., Massillon, OH, USA). According to the FXYD6 mRNA sequence published by GeneBank (ID: NM-022003), a pair of primers was designed to amplify the FXYD6 functional region sequence: the upstream primer was 5′-GAATTCAGTGCAGCTGAAAAGGAG-3′, and the downstream primer was 5′-CTCGAGTCAGTTCTCTGCTTTCTGG-3′. Meanwhile, the glyceraldehyde-3-phosphate dehydrogenase (GAPDH) gene used as a reference for normalization was also amplified. Its upstream primer was 5′-GGTGAAGGTCGGAGTCAACG-3′, and the downstream primer was 5′-CAAAGTTGTCATGGATGHACC-3′. Each PCR reaction contained 1 µL RT product, 20 pM of each primer, 800 µM dNTP (200 µM each), 1 unit Taq DNA polymerase, and nuclease-free H_2_O up to 50 µL. Reactions were performed on a T100 Thermal Cycler (Bio-Rad, Hercules, CA, USA). Amplification was conducted under the following thermal cycling conditions: 5 min at 95°C; 36 cycles of amplification consisting of 30 s at 95°C, 30 s at 53°C, and 30 s at 72°C; and a final extension at 72°C for 10 min. For analysis, the product was loaded in agarose gel, using GAPDH as a reference standard, electrophoresed, and photographed by a UV gel imaging analyzer (Bio-Rad, Hercules, CA, USA). The integral optical density (IOD) of the PCR product strip was detected using the Quantity One software (v4.6.2; Bio-Rad, Hercules, CA, USA). The ratio of the FXYD6 IOD to the corresponding GAPDH IOD was taken as the relative amount of FXYD6 mRNA in the tissues.

### 2.3 Immunohistochemistry

The HBV-related HCC with cirrhosis tissues, cirrhosis tissues adjacent to the carcinoma, and normal liver tissues were fixed in 10% formalin, embedded in paraffin, and serially sectioned at 4 µm. Each section was incubated at 60°C, deparaffinized, and rehydrated. Antigen retrieval was performed by placing slides in 10 mmol/L sodium citrate buffer (pH 6.0) and microwave treatment for 15 min. Then, the slides were allowed to cool down naturally in the buffer to room temperature. Endogenous peroxidase activity was eliminated by incubation in methanol with 0.3% H_2_O_2_ for 30 minutes, followed by washing with phosphate-buffered saline (PBS). Following non-specific reactions elimination with 5% normal horse serum for 1 h, the sections were incubated with mouse anti-human monoclonal FXYD6 prepared by Gao et al. [8] overnight at 4°C in a moist chamber. After washing in PBS, the sections were incubated with a biotinylated horse anti-mouse IgG antibody (ZB-2020; ZSGB-BIO, China) for 40 min at 37°C, washed again in PBS, and inculcated in horseradish peroxidase streptavidin (ZB-2404; ZSGB-BIO) for 40 min. The peroxidase reaction was developed in freshly prepared 3,3′-diaminobenzidine solutions (ZLI-9017; ZSGB-BIO), which was observed under a microscope (BX531; Olympus, Tokyo). Then, the sections were rinsed in water and counterstained using hematoxylin, dehydrated in ethanol, and mounted with xylene-based mounting medium. For negative controls, PBS was used instead of the FXYD6 monoclonal antibody under the same conditions.

The expression of FXYD6 in the tissues was evaluated with whole slide scanning under low magnification (×40) and then confirmed under high magnification (×100 and ×400). An immunoreactivity scoring system was applied. The extent of stained cells was given as follows: 0–5% = 0, 6–25% = 1, 26–50% = 2, 51–75% = 3, and 75–100% = 4. The intensity of color staining was defined by the following parameters: colorless, 0; whitish yellow, 1; yellow, 2; and brown, 3. The final immunoreactive score was determined by multiplying the intensity and extent of positivity scores of stained cells, with a minimum score of 0 and a maximum score of 12. The threshold for differentiating between final positive and negative immunostaining was set at 4 for interpretation. A negative staining was classified as having an immunostaining score of 0–3, whereas a positive staining was classified as having an immunostaining score of 4–12.

The slides were examined independently by two pathologists blinded to both clinical and pathological data, and all discrepancies were resolved by joint review of the slides in question.

### 2.4 Statistical analysis

Measurement data were expressed as mean ± SD, and statistical differences were determined by the independent samples *t* test. Classification data were shown as rate, and the comparison was analyzed by the chi-square test. These statistical analyses were all performed through SPSS software (v17.0; SPSS Inc., Chicago, IL, USA). For all analyses, *p*-values were two-tailed, and *p* < 0.05 was considered to indicate a statistically significant difference.

## 3 Results

### 3.1 FXYD6 mRNA expression is markedly upregulated in HCC

**Figure 1:**
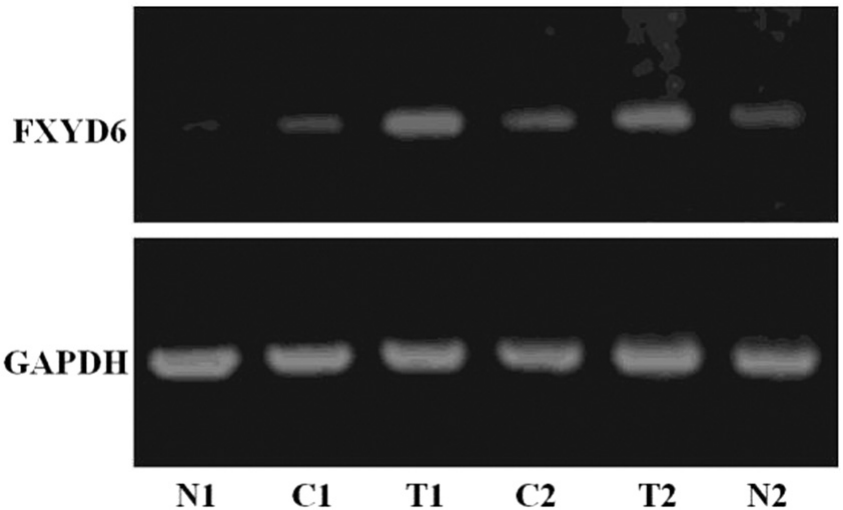
The expression of FXYD6 mRNA in HCC with HBV-associated cirrhosis, paracancerous cirrhosis, and normal liver tissues. There was high expression of FXYD6 mRNA in HCC and low expression of FXYD6 mRNA in cirrhosis and normal liver tissues. N: normal live tissues, C: cirrhosis tissues, and T: HCC with HBV-related cirrhosis tissues.

The results of FXYD6 mRNA expression in HCC, cirrhosis, and normal liver tissues by semi-qualitative RT-PCR are shown in Figure 1 and Table 1. The relative IOD of FXYD6 mRNA expression was significantly higher in 35 HCC than that in 30 cirrhotic tissues distal to HCC (0.4461 ± 0.0344 vs. 0.2887 ± 0.0176; *t* = 23.723; *p* = 0.000) and significantly higher than that in 10 normal liver tissues without HBV infection (0.4461 ± 0.0344 vs. 0.2781 ± 0.0422; *t* = 12.959; *p* = 0.000). But there was no statistical difference between the relative IOD of FXYD6 mRNA expression in 30 cirrhosis tissues and that in 10 normal liver tissue (0.2887 ± 0.0176 vs. 0.2781 ± 0.0422; *t* = 0.771; *p* = 0.458).

**Table 1:** Relative IOD results of FXYD6 mRNA by semiquantitative RT-PCR

Tissue type	*N*	IOD value	SD	*t*-Value	*p-*Value
Normal liver	10	0.2781	0.0422	0.771^a^	0.458^a^
Cirrhosis	30	0.2887	0.0176	23.723^b^	0.000^b^
HCC	35	0.4461	0.0344	12.959^c^	0.000^c^

a Normal liver vs. cirrhosis.

b HCC vs. cirrhosis.

c HCC vs. normal liver.

### 3.2 FXYD6 protein is highly expressed in HCC

We examined FXYD6 protein immunohistochemically in 52 HBV-related HCC with cirrhosis tissues, 28 distal non-cancerous cirrhosis tissues, and 15 normal liver tissues. This study showed that there was negative immunostaining of FXYD6 in most cirrhosis tissues and in most normal liver tissues. FXYD6 negative reactivity was observed in 10/15 (66.7%) and 18/28 (64.3%) in normal liver slides and cirrhosis slides, respectively (Table 2). However, the positive expression rate of FXYD6 was 4/7 (57.1%) for well-differentiated HCC, 20/24 (83.3%) for moderately differentiated HCC, and 17/21 (81.0%) for poorly differentiated HCC. In all, the positive expression rate of FXYD6 in HCC was 41/52 (78.8%), which was significantly higher than that in normal tissues 5/15 (33.3%) or that in cirrhosis tissues 10/28 (35.7%).

**Table 2:** FXYD6 protein expression in HCC with HBV-related cirrhosis, paracancerous cirrhosis, and normal liver tissues

Tissue type	*n*	FXYD6 expression		*X* ^2^	*p*-Value
		Negative (%)	Positive (%)		
Normal liver	15	10 (66.7)	5 (33.3)	0.024^a^	0.876^a^
Cirrhosis	28	18 (64.3)	10 (35.7)	14.651^b^	0.000^b^
HCC	52	11 (21.2)	41 (78.8)	9.191^c^	0.002^c^

a Normal liver vs. cirrhosis.

b HCC vs. cirrhosis.

c HCC vs. normal liver.

### 3.3 Expression of FXYD6 correlates with MVI, pathological stage, and early recurrence

**Figure 2:**
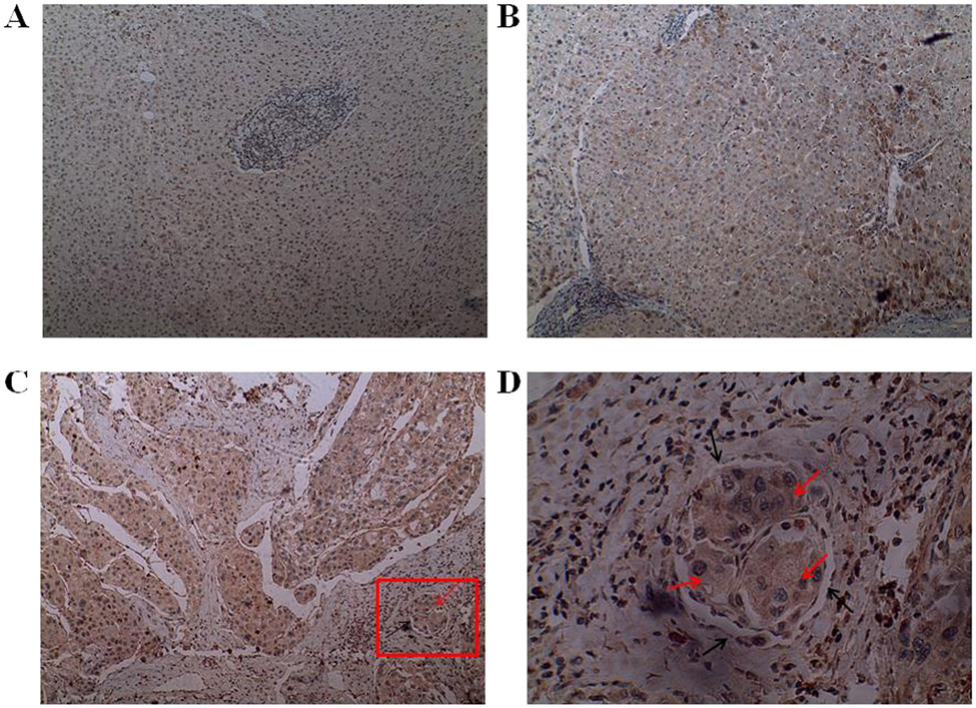
Representative immunohistochemical staining in normal liver, cirrhosis, and HCC. (A) Negative signal of FXYD6 was detected in normal liver tissues. (B) Most hepatocytes negatively expressed FXYD6, but a few hepatocytes next to portal area positively expressed FXYD6 in cirrhosis. (C and D) Positive expression of FXYD6 was found in moderately differentiated HCC, and FXYD6 was also highly expressed in the tumor cells (the red arrow) infiltrating the microvessel (the black arrow). Magnification (A, B, and C) ×100 and (D) ×400.

The expression level of FXYD6 in HCC was related to MVI and pathological stage. We examined the expression of FXYD6 protein in HCC, cirrhosis, and normal liver and found that the protein is mainly located in the cytoplasm but not in the nucleus as shown in Figure 2. The correlation between FXYD6 expression and various clinicopathological factors was also analyzed. As shown in Table 3, FXYD6 protein expression was positively correlated with MVI, and the positive expression rate of FXYD6 in HCC with MVI (20/21, 95.2%) was higher than that without MVI (21/31, 67.7%). In our studies, the FXYD6 expression was also associated with the pathological stage. Increased FXYD6 expression was found to significantly correlate with the degree of pathological stage of HCC. The positive expression rate of FXYD6 in HCC with pathological stages I–II (22/33, 66.7%) was significantly lower than that with pathological stage III (18/19, 94.7%). But there was no significant correlation between the increased expression of FXYD6 and other clinicopathological factors, including gender (*X* ^2^ = 0.000, *p* = 1.000), age (*X* ^2^ = 0.002, *p* = 0.968), histological differentiation (*X* ^2^ = 2.322, *p* = 0.313), tumor diameter (*X* ^2^ = 1.610, *p* = 0.204), tumor number (*X* ^2^ = 0.983, *p* = 0.321), integrity of tumor capsule or not (*X* ^2^ = 2.028, *p* = 0.154), and AFP concentration in serum (*X* ^2^ = 0.000, *p* = 1.000).

**Table 3:** Correlation between FXYD6 protein expression and clinicopathological variables in patients with HCC

Variables	*n*	FXYD6 expression		*X* ^2^	*p*-Value
		Negative (%)	Positive (%)		
Gender				0.000	1.000
Male	44	9 (20.5)	35 (79.5)		
Female	8	2 (25.0)	6 (75.0)		
Age (years)				0.002	0.968
<60	31	6 (19.4)	25 (80.6)		
≥60	21	5 (23.8)	16 (76.2)		
Differentiation				2.322	0.313
Well	7	3 (42.9)	4 (57.1)		
Moderately	24	4 (16.7)	20 (83.3)		
Poor	21	4 (19.0)	17 (81.0)		
Tumor diameter (cm)				1.610	0.204
≤5	22	7 (31.8)	15 (68.2)		
>5	30	4 (13.3)	26 (86.7)		
Tumor number				0.983	0.321
Single	40	11 (27.5)	29 (72.5)		
Multiple	12	1 (8.3)	11 (91.7)		
Tumor capsule				2.028	0.154
Integrity	31	4 (12.9)	27 (87.1)		
No integrity	21	7 (33.3)	14 (66.7)		
MVI				4.146	0.042
Positive	21	1 (4.8)	20 (95.2)		
Negative	31	10 (32.3)	21 (67.7)		
Pathological stage				3.888	0.049
I–II	33	11 (33.3)	22 (66.7)		
III	19	1 (5.3)	18 (94.7)		
AFP (ng/mL)				0.000	1.000
<400	38	8 (21.1)	30 (78.9)		
≥400	14	3 (21.4)	11 (78.6)		

Well = well-differentiated HCC, mod = moderately differentiated HCC, poor = poorly differentiated HCC.

We also statistically analyzed the relationship between FXYD6 protein expression and early recurrence in postoperative patients with HCC. We followed up these 52 HCC patients who underwent curative hepatectomy and received no treatment before the HCC recurrence was discovered and found that the expression level of FXYD6 in HCC was related to early recurrence. The recurrence rate within 1 year in the high FXYD6 protein expression group was obviously higher than that in the low FXYD6 protein expression group (22/41, 53.7% vs. 2/11, 18.2%; *X* ^2^ = 4.392, *p* = 0.036).

## 4 Discussion

HCC with a high incidence rate in males is considered to be a highly fatal disease due to the concealed onset of HCC, early intrahepatic metastasis through portal venous, and lack of effective therapy [11]. Risk factors for HCC include viral infections caused by HBV and/or hepatitis C virus, alcoholic liver disease, nonalcoholic fatty liver, and genetically inherited metabolic disease [12]. In Asia, chronic HBV infection is the leading cause of cirrhosis, which is a chronic progressive liver disease characterized by diffuse fibrosis of the liver parenchyma and pseudolobular formation and eventually leads to hepatocellular carcinogenesis [13].

Radical hepatectomy and liver transplantation are the most effective treatments for the malignant tumor nowadays. However, MVI, one of the invasive features of HCC, mainly occurs in the small branch of the portal vein next to the cancer and is an independent factor for intrahepatic and distant metastases [14]. It is also related to early recurrence after curative hepatectomy [15]. Therefore, searching for proteins associated with MVI of HCC may provide new ideas for reducing intrahepatic metastasis, early recurrence, and improving the prognosis of HCC patients.

The FXYD protein family was characterized by a signature sequence which contained an FXYD motif and three other conserved amino acid residues and have seven members in mammals [16]. The family protein is a regulator of Na, K-ATPase, which plays an important role in the formation and maintenance of sodium and potassium ion transmembrane concentration gradients [17]. Meanwhile, members of the FXYD protein family such as FXYD3 and FXYD5 play an important role in the pathogenesis of numerous malignant tumors and are used as indicators for the biological characteristics and prognostic factors of certain tumors [18,19,20,21]. FXYD6 cDNA was first cloned in a rat hippocampus library by Yamaguchi [22], who named the encoded protein phosphohippolin, and FXYD6 protein was expressed highly in neurons and associated with neuronal cell development and synaptic signal transduction in a later research [6]. Shiina *et al.* [5] had demonstrated that the formation of neuronal networks was significantly inhibited through downregulation of FXYD6 protein. The FXYD6 gene is located at 11q23.3 in a schizophrenia-linked segment and is associated with schizophrenia [23,24]. FXYD6 is also a tumor-associated protein and plays an important role in various tumors. FXYD6 is differentially expressed between osteosarcoma and normal control tissues [25]. Moreover, it is a binding target of microRNA-137 and miR-372-3p, and the protein is involved in the proliferation and migration of the osteosarcoma cells [9,10]. Furthermore, we previously reported that FXYD6 protein was upregulated in cholangiocarcinoma and was associated with tumor differentiation. It was highly expressed in well and moderately differentiated cholangiocarcinoma, but lowly expressed in poorly differentiated cholangiocarcinoma [7]. According to a preliminary study by Lu et al. [26], patients, who had colorectal carcinoma synchronous liver metastasis with high expression of FXYD6, were sensitive to the FOLFOX4 chemotherapy regimen by using DNA microarray analysis. Our previous study also found that the expression level of FXYD6 protein in HCC, thyroid carcinoma, and colon carcinoma was higher than that in the corresponding normal tissues by conducting a immunohistochemical screen on a commercial human tissue array and that FXYD6 protein was expressed in some HCC cell lines and has contributed to the proliferation and metastasis of HepG2 cells by upregulating the α1 subunit of Na, K-ATPase in vitro and activating the downstream Src-ERK signaling components [8]. In this study, the FXYD6 mRNA was semiquantitatively detected in 35 fresh HCC, 30 fresh corresponding paracancerous cirrhosis tissues, and 10 fresh liver tissues; the protein was also examined in 52 HCC with HBV infection, 28 cirrhosis tissues, and 15 normal liver tissues; and the clinicopathological significance of FXYD6 protein expression in HCC was also analyzed. The results showed that FXYD6 was significantly associated with HCC, and FXYD6 mRNA and protein were upregulated in HCC compared with normal liver tissue and HBV-related cirrhosis, indicating that FXYD6 may be a new biomarker and therapeutic target for HCC. But the expression difference of FXYD6 mRNA and protein between normal liver tissue and cirrhosis was not obvious, indicating that the FXYD6 protein might not participate in the process of transforming normal liver tissue into cirrhosis with HBV infection, but acted as an essential factor of the hepatocellular carcinogenesis in cirrhosis.

Additionally, the FXYD6 protein expression was also observed to be positively associated with MVI and pathological stage in HCC. As shown in Table 3, the positive expression rate of FXYD6 in the group without MVI was significantly lower than that in the group with MVI (67.2% vs. 95.2%; *p* = 0.042). The positive rate of FXYD6 expression increased with a higher pathological stage. The positive expression rate of FXYD6 in the stage III was obviously higher than that in stages I–II (94.7% vs. 66.7%; *p* = 0.049).

In this study, we followed up the HCC patients undergoing radical surgery for 1 year and found that there was a statistically higher recurrence rate in the HCC patients with the positive expression of FXYD6 protein than that with the negative expression of FXYD6 protein (53.7% vs. 18.2%; *p* = 0.036). The result indicated that the higher the expression of FXYD6 in patients with HCC, the more likely the HCC relapsed after operation. Radical surgery is currently the most effective therapy for HCC, but due to high recurrence rate, there is no breakthrough in the overall prognosis of HCC. Since FXYD6 could promote HCC cell invasion and proliferation [8], we assumed that increased level of FXYD6 protein may contribute to early HCC recurrence through promoting tumor cells MVI which, in turn, facilitates intrahepatic dissemination of the tumor and thus upgrading the pathological stage. However, this hypothesis requires further study to be confirmed. The result suggested that FXYD6 might be a therapeutic target for decreasing intrahepatic metastasis in HCC and a poor prognosis of the HCC patients.

The significant differences in FXYD6 expression between HCC tissues and the corresponding cirrhosis indicated that the protein might be associated with carcinogenesis of hepatocyte in cirrhosis. As HCC with frequent FXYD6 expression had a high recurrence rate, the protein might be involved in HCC progression. The mechanism of FXYD6 overexpression in HCC was not understood and required further exploration.

The insufficiency of this study was that the expression of FXYD6 was not detected in HCC caused by other factors except HBV-related cirrhosis. Because the cirrhosis with HBV infection was the leading factor of HCC in our country and the sample size was small in the study, only the expression of FXYD6 protein and mRNA in HBV-related HCC with cirrhosis was studied. In addition, statistical analysis of the correlation between FXYD6 expression and survival was not performed because the HCC patient’s treatment after tumor recurrence was different.

## 5 Conclusion

In summary, FXYD6 protein was highly expressed in HCC and associated with MVI, pathological stage, and early recurrence of the tumor. The FXYD6 protein might serve as a novel target for the diagnosis and treatment of HCC.

## Abbreviations

FXYD6: FXYD domain containing ion transport regulator 6
GAPDH: glyceraldehyde-3-phosphate dehydrogenase
HBV: hepatitis B virus
HCC: hepatocellular carcinoma
IOD: integral optical density
MVI: microvascular invasion
PBS: phosphate-buffered saline
RT-PCR: reverse transcription polymerase chain reaction

## References

[1] Allemani C, Matsuda T, Di Carlo V, Harewood R, Matz M, Nikšić M, et al. Global surveillance of trends in cancer survival 2000–14 (CONCORD-3): analysis of individual records for 37,513,025 patients diagnosed with one of 18 cancers from 322 population-based registries in 71 countries. Lancet. 2018;391(10125):1023–75. 10.1016/S0140-6736(17)33326-3 PMC5879496 29395269

[2] Baecker A, Liu X, La Vecchia C, Zhang ZF. Worldwide incidence of hepatocellular carcinoma cases attributable to major risk factors. Eur J Cancer Prev. 2018;27(3):205–12. 10.1097/CEJ.0000000000000428 PMC5876122 29489473

[3] Tokumitsu Y, Sakamoto K, Tokuhisa Y, Matsui H, Matsukuma S, Maeda Y, et al. A new prognostic model for hepatocellular carcinoma recurrence after curative hepatectomy. Oncol Lett. 2018; 15(4):4411–22 10.3892/ol.2018.7821 PMC5844062 29556288

[4] Miyashita T, Akiyama K, Inamoto R, Matsubara A, Nakagawa T, Yamaguchi F, et al. Presence of FXYD6 in the endolymphatic sac epithelia. Neurosci Lett. 2012; 513(1):47–50. 10.1016/j.neulet.2012.02.005 22343024

[5] Shiina N, Yamaguchi K, Tokunaga M. RNG105 deficiency impairs the dendritic localization of mRNAs for Na+/K+ ATPase subunit isoforms and leads to the degeneration of neuronal networks. J Neurosci. 2010;30(38):12816–30. 10.1523/JNEUROSCI.6386-09.2010 PMC6633578 20861386

[6] Biesemann C, Grønborg M, Luquet E, Wichert SP, Bernard V, Bungers SR, et al. Proteomic screening of glutamatergic mouse brain synaptosomes isolated by fluorescence activated sorting. EMBO J. 2014;33(2):157–70. 10.1002/embj.201386120 PMC3989609 24413018

[7] Chen X, Sun M, Hu Y, Zhang H, Wang Z, Zhou N, et al. FXYD6 is a new biomarker of cholangiocarcinoma. Oncol Lett. 2014;7(2):393–8. 10.3892/ol.2013.1727 PMC3881923 24396454

[8] Gao Q, Chen X, Duan H, Wang Z, Feng J, Yang D, et al. FXYD6: a novel therapeutic target toward hepatocellular carcinoma. Protein Cell. 2014;5(7):532–43. 10.1007/s13238-014-0045-0 PMC4085285 24715268

[9] Li ZM, Zhang HY, Wang YX, Wang WB. MicroRNA-137 is downregulated in human osteosarcoma and regulates cell proliferation and migration through targeting FXYD6. J Drug Target. 2016; 24(2):102–10. 10.3109/1061186X.2015.1057149 26302771

[10] Xu SY, Xu PF, Gao TT. MiR-372-3p inhibits the growth and metastasis of osteosarcoma cells by targeting FXYD6. Eur Rev Med Pharmacol Sci. 2018;22(1):62–9. 10.26355/eurrev_201801_14101 29364472

[11] Hartke J, Johnson M, Ghabril M. The diagnosis and treatment of hepatocellular carcinoma. Semin Diagn Pathol. 2017;34(2):153–9. 10.1053/j.semdp.2016.12.011 28108047

[12] Fattovich G, Stroffolini T, Zagni I, Donato F. Hepatocellular carcinoma in cirrhosis: incidence and risk factors. Gastroenterology. 2004;127(5 suppl 1):S35–S50. 10.1053/j.gastro.2004.09.014 15508101

[13] Croaqh CM, Lubel JS. Natural history of chronic hepatitis B: phases in a complex relationship. World J Gastroenterol. 2014;20(30):10395–404. 10.3748/wjg.v20.i30.10395 PMC4130846 25132755

[14] Shimada S, Kamiyama T, Yokoo H, Orimo T, Wakayama K, Einama T, et al. Clinicopathological characteristics of hepatocellular carcinoma with microscopic portal venous invasion and the role of anatomical liver resection in these cases. World J Surg. 2017;41(8):2087–94 10.1007/s00268-017-3964-0 28271260

[15] Matsumoto T, Kubota K, Aoki T, Iso Y, Kato M, Shimoda M. Clinical impact of anatomical liver resection for hepatocellular carcinoma with pathologically proven portal vein invasion. World J Surg. 2016;40(2):402–11. 10.1007/s00268-015-3231-1 26306893

[16] Franzin CM, Gong XM, Teriete P, Marassi FM. Structures of the FXYD regulatory proteins in lipid micelles and membranes. J Bioenerg Biomembr. 2007;39(5–6):379–83. 10.1007/s10863-007-9105-y PMC2917602 18000745

[17] Clausen MV, Hilbers F, Poulsen H. The structure and function of the Na, K-ATPase isoforms in health and disease. Front Physiol. 2017;8:371. 10.3389/fphys.2017.00371 PMC5459889 28634454

[18] Xue Y, Lai L, Lian W, Tu X, Zhou J, Dong P, et al. SOX9/FXYD3/Src axis is critical for ER+ breast cancer stem cell function. Mol Cancer Res. 2019;17(1):238–49 10.1158/1541-7786.MCR-18-0610 30206184

[19] Wang LJ, Li QJ, Le Y, Ouyang HY, He MK, Yu ZS, et al. Prognostic significance of sodium-potassium ATPase regulator, FXYD3, in human hepatocellular carcinoma. Oncol Lett. 2018;15(3):3024–30 10.3892/ol.2017.7688 PMC5778849 29435033

[20] Raman P, Purwin T, Pestell R, Tozeren A. FXYD5 is a marker for poor prognosis and a potential driver for metastasis in ovarian carcinomas. Cancer Inform. 2015;14:113–9. 10.4137/CIN.S30565 PMC4603440 26494976

[21] Lubarski-Gotliv I, Dey K, Kuznetsov Y, Kalchenco V, Asher C, Garty H. FXYD5 (dysadherin) may mediate metastatic progression through regulation of the β-Na+-K+-ATPase subunit in the 4T1 mouse breast cancer model. Am J Physiol Cell Physiol. 2017;313(1):C108–17 10.1152/ajpcell.00206.2016 28515087

[22] Yamaguchi F, Yamaguchi K, Tai Y, Sugimoto K, Tokuda M. Molecular cloning and characterization of a novel phospholemman-like protein from rat hippocampus. Brain Res Mol Brain Res. 2001;86(1–2):189–92. 10.1016/s0169-328x(00)00213-8 11165386

[23] Zhong N, Zhang R, Qiu C, Yan H, Valenzuela RK, Zhang H, et al. A novel replicated association between FXYD6 gene and schizophrenia. Biochem Biophys Res Commun. 2011;405(1):118–21. 10.1016/j.bbrc.2011.01.005 21216238

[24] Ito Y, Nakamura Y, Takahashi N, Saito S, Aleksic B, Iwata N, et al. A genetic association study of the FXYD domain containing ion transport regulator 6 (FXYD6) gene, encoding phosphohippolin, in susceptibility to schizophrenia in a Japanese population. Neurosci Lett. 2008;438(1):70–5. 10.1016/j.neulet.2008.04.010 18455306

[25] Yang Z, Chen Y, Fu Y, Yang Y, Zhang Y, Chen Y, et al. Meta-analysis of differentially expressed genes in osteosarcoma based on gene expression data. BMC Med Genet. 2014;15:80. 10.1186/1471-2350-15-80 PMC4109777 25023069

[26] Lu X, Pan J, Li S, Shen S, Chi P, Lin H, et al. Establishment of a predictive genetic model for estimating chemotherapy sensitivity of colorectal cancer with synchronous liver metastasis. Cancer Biother Radiopharm. 2013;28(7):552–8. 10.1089/cbr.2012.1431 PMC3741425 23721165

